# INTERGENERATIONAL RELATIONSHIPS AMONG OLDER ADULTS AND GRANDCHILDREN: SUPPORTIVE AND CONFLICTUAL RELATIONSHIPS

**DOI:** 10.1093/geroni/igz038.637

**Published:** 2019-11-08

**Authors:** Sofia von Humboldt, Ana Monteiro, Isabel Leal

**Affiliations:** 1 William James Center for Research, ISPA - Instituto Universitario, Lisbon, Portugal; 2 ISPA - Instituto Universitario, Lisbon, Portugal

## Abstract

Objectives: To analyze how older adults conceptualize these intergenerational relationships. Methods: In this qualitative study, in-depth interviews were carried out with 316 older adults, aged 65-102, from three different nationalities who lived at home. Verbatim transcripts were examined. Results: Data analysis generated six themes representing intergenerational relationships: affection and reward; interest and integration; grandparent-grandchild interaction quality; privacy and boundaries definition; provision of support; and obligation of providing childcare, on two dimensions of ambivalence concerning their intergenerational relationships (supportive and conflictual). Conclusions: The empirical findings from this research indicate how ambivalence in intergenerational relationships is experienced by older adults and stress the contradictory expectations of older adults with grandchildren. Keywords: Ambivalence; conflict; intergenerational relationships; older adults; support.

